# Design and experimental validation of OPERA_MET-A panel for deep methylation analysis by next generation sequencing

**DOI:** 10.3389/fonc.2022.968804

**Published:** 2022-08-11

**Authors:** Federico Pio Fabrizio, Stefano Castellana, Flavia Centra, Angelo Sparaneo, Mario Mastroianno, Tommaso Mazza, Michelina Coco, Domenico Trombetta, Nicola Cingolani, Antonella Centonza, Paolo Graziano, Evaristo Maiello, Vito Michele Fazio, Lucia Anna Muscarella

**Affiliations:** ^1^ Laboratory of Oncology, Fondazione IRCCS, Scientific Institute for Research and Health Care Casa Sollievo della Sofferenza, San Giovanni Rotondo, Italy; ^2^ Unit of Bioinformatics, Fondazione IRCCS Casa Sollievo della Sofferenza, San Giovanni Rotondo, Italy; ^3^ Scientific Direction, Fondazione IRCCS Casa Sollievo della Sofferenza, San Giovanni Rotondo, Italy; ^4^ Unit of Pathology, Fondazione IRCCS Casa Sollievo della Sofferenza, San Giovanni Rotondo, Italy; ^5^ Unit of Oncology, Fondazione IRCCS Casa Sollievo della Sofferenza, San Giovanni Rotondo, Italy; ^6^ Laboratory of Molecular Medicine and Biotechnology, University Campus Bio-Medico of Rome, Rome, Italy; ^7^ Institute of Translational Pharmacology, National Research Council of Italy (CNR), Rome, Italy

**Keywords:** cancer, driver gene, biomarker, methylation, next generation sequencing

## Abstract

DNA methylation is the most recognized epigenetic mark that leads to a massive distortion in cancer cells. It has been observed that a large number of DNA aberrant methylation events occur simultaneously in a group of genes, thus providing a growth advantage to the cell in promoting cell differentiation and neoplastic transformation. Due to this reason, methylation profiles have been suggested as promising cancer biomarkers. Here, we designed and performed a first step of validation of a novel targeted next generation sequencing (NGS) panel for methylation analysis, which can simultaneously evaluate the methylation levels at CpG sites of multiple cancer-related genes. The OPERA_MET-A methylation panel was designed using the Ion AmpliSeq™ technology to amplify 155 regions with 125-175 bp mean length and covers a total of 1107 CpGs of 18 cancer-related genes. The performance of the panel was assessed by running commercially available fully methylated and unmethylated control human genomic DNA (gDNA) samples and a variable mixture of them. The libraries were run on Ion Torrent platform and the sequencing output was analyzed using the “methylation_analysis” plugin. DNA methylation calls on both Watson (W) and Crick (C) strands and methylated:unmethylated ratio for each CpG site were obtained. Cell lines, fresh frozen and formalin-fixed paraffin-embedded (FFPE) lung cancer tissues were tested. The OPERA_MET-A panel allows to run a minimum of 6 samples/530 chip to reach an observed mean target depth ≥2,500X (W and C strands) and an average number of mapped reads >750,000/sample. The conversion efficiency, determined by spiking-in unmethylated Lambda DNA into each sample before the bisulfite conversion process, was >97% for all samples. The observed percentage of global methylation for all CpGs was >95% and <5% for fully methylated and unmethylated gDNA samples, respectively, and the observed results for the variable mixtures were in agreement with what was expected. Methylation-specific NGS analysis represents a feasible method for a fast and multiplexed screening of cancer patients by a high-throughput approach. Moreover, it offers the opportunity to construct a more robust algorithm for disease prediction in cancer patients having a low quantity of biological material available.

## 1 Introduction

DNA methylation is one of the most largely investigated epigenetic footprints, due to its link with several diseases as well as cancers and autoimmune or genetic disorders (1, 2). Of particular interest is the role of DNA methylation at cytosine residues by the addition of a methyl group (5-mC), most frequently at the CpG (cytosine-phosphate-guanine) dinucleotide motif of mammalian genomes. The fluctuation of 5-mC level is generally associated with variation in the genes expression levels and contributes in many cases to the definition of clinical phenotypes, as well as representing in some clinical contexts a useful diagnostic biomarker in guiding therapeutical choices (3, 4).

In tumors, the epigenome alteration is linked to the neoplastic transformation, cancer progression and invasion, and encouraged a large number of studies focused on discovering predictive and prognostic power of the methylation status at CpG sites (5). To date, the CpG methylation changes represent an attractive source of biomarkers that could have a significant impact on both early and advanced tumors management. Moreover, methyl CpG sites could arise during therapy resistance in patients, so they could represent a new option for the longitudinal monitoring of the neoplastic evolution. Many scientific findings originate from managing large long-existing available datasets and, in this context, the aberrant methylation of the CpGs located at the promoter regions of tumor suppressor genes is gaining prominence (6–8). By contrast, only few large-scale studies have been focused on the role and prognostic impact on cancer of different methylation patterns across the genes, such as intragenic or gene body methylation, that may have a different role in the transcriptional regulation and efficiency of genes machinery (9, 10).

The translation of all this epigenetic knowledge in clinical practice is not complete, mainly due to the lack of high-throughput and quantitatively accurate approaches that can rapidly profile poor quality and quantity of DNA obtained by tumor tissue biopsies. Pyrosequencing and bisulfite-cloning/sequencing are the most widely used methods for low-cost analyses to measure the methylation level at single CpGs of genes in daily practice with reasonable quantitative accuracy. Although highly useful, the limitations of these techniques include samples consumption, short-read length, and low sample throughput (11).

In this methodology paper, we designed and experimentally validated a customized methylation panel for NGS analysis (OPERA_MET-A panel), to scan relevant CpG sites in 155 regions of 18 cancer-related genes mainly involved in the NRF2/KEAP1 pathway and immunotherapy. gDNA from FFPE, frozen tissues and cell lines were tested to simultaneously evaluate their density and average methylation levels for all or single targeted CpG sites. Finally, using this NGS approach, we obtained details about the strand-specific specificity of CpG methylation in targeted regions of the selected genes.

## 2 Material and methods

### 2.1 Biological samples selection

Three different types of biological samples were used to validate the OPERA_MET-A panel: n.3 lung paired tumor/non-neoplastic FFPE tissues (830T/N, 881T/N, 889T/N), n.3 lung tumors optimal cutting temperature compound (OCT) embedded (435T, 475T, 495T), n.2 lung cell lines (tumor A459 and normal MRC5) were used. Tissues were collected from anonymous patients, according to the guidelines of the Local Ethical Committee of IRCCS Casa Sollievo della Sofferenza Hospital, Italy, whereas cell lines were purchased from the American Type Culture Collection (ATCC, Manassas, VA, USA). A commercially available fully methylated (>95%, CpGenome Universal Methylated DNA, Millipore, Chemicon) and unmethylated (<5%, CpGenome Universal Unmethylated DNA, Chemicon) genomic gDNA with four mixtures (~25%, ~50%, ~75% and ~90%) were used as positive and negative controls to the optimal DNA conversion and library preparation.

### 2.2 gDNA extraction and sodium bisulfite conversion

gDNA was extracted from cell lines and fresh OCT embedded tissues by using the standard phenol-chloroform procedure (12), whereas 10 μm FFPE sections were extracted using the GeneRead DNA FFPE kit (Qiagen, MD, USA), following the manufacturer’s instruction. Before starting the bisulfite conversion, 0,33% of Unmethylated Lambda DNA (Promega) was added to each single gDNA in order to estimate bisulfite conversion efficiency during samples treatment. For each sample and control mixture (methylated/unmethylated DNA), a minimum of 250ng of gDNA extracted was treated by using Epitect Bisulfite kit (Qiagen) (13), to ensure a minimum of 100ng of converted gDNA recovery for libraries preparation. The quantification of both extracted gDNA and bisulfite treated gDNA was performed using Qubit^®^ ssDNA Assay Kit on Qubit™ 3.0 Fluorometer (Thermo Fisher, Life Scientific).

### 2.3 NGS workflow

#### 2.3.1 Ion AmpliSeq™ methylation panel design

The OPERA_MET-A panel was designed in collaboration with the Ion AmpliSeq custom design team through the Ion AmpliSeq White Glove Service (Thermo Fisher Scientific); it includes multiple informative and challenging genes regions also tracked by the Infinium Human Methylation 450 BeadChip (Illumina Inc.) or annotated as having a prognostic value in peer-reviewed scientific papers. Specifically, the OPERA_MET-A panel allows the simultaneous quantification and analysis of 155 genomic regions, each of them covered by amplicons with a size range of 125-175bp length (amplicons designed for both Watson and Crick strands) and covers 1107 CpGs located in 18 cancer-related genes (*KEAP1, SPARC, PD-L1/CD274, PD-1/CD279, HAR1B, TMPRSS4, RASSF1A, HOXA9, NFE2L2, GPX2, PGD, TXNRD1, GCLC, AKR1C2, SRXN1, ABCC2, PTEN, CDKN2A/P16*, GRCh37/hg19 release). Details about the CpG sites included in the methylation NGS panel were fully listed in **Supplemental Table 1**.

#### 2.3.2 Library preparation and sequencing

Targeted amplifications were performed starting from a standard quantity of bisulfite converted gDNA for each multiplex PCR amplification. Amplicons were generated using the two primer pools of the OPERA_MET-A panel, following the manufacturer’s recommendation for the Ion AmpliSeq Library Kit Plus for Bisulfite methylation library production (Thermo Fisher Scientific). Briefly, 2 μL of 5X Ion AmpliSeq™ HiFi mix, 2 μL of 5X Ion AmpliSeq™ of primers pools and 100ng of bisulfite converted gDNA were mixed and amplified following temperature conditions to achieve DNA target amplification. Then, primer sequences were partially digested by adding 2 μL of FuPa Reagent and loaded in a thermal cycler under user guide conditions. Each library was labeled with a single Ion Xpress™ barcode and Ion P1 adapter (Thermo Fisher Scientific) by adding 4 μL of Switch Solution, 2 μL of diluted barcode and 2 μL of DNA Ligase to the reaction mixture, following the temperature conditions recommended by the manufacturer. Finally, the Agencourt AMPure XP bead (Beckman Coulter, Brea, CA, USA) was used to complete three rounds of purifications on a magnet rack and 50 µL of Low TE was added to elute the library. A 100-fold dilution of purified and amplified libraries was quantified using the Ion Library TaqMan quantitation kit (Thermo Fisher Scientific) in a 7900 Real-Time PCR System (Thermo Fisher Scientific), following standard qPCR cycling. Library profiles were checked by capillary electrophoresis using the High Sensitivity D1000 ScreenTape on Tape Station 2200 (Agilent Technologies), and equimolar concentrations of samples libraries were pooled. 40pM of manually pooled normalized libraries were used for template preparation and Ion 530™ chip loading on the Ion Chef Instrument (Thermo Fisher Scientific) using the Ion 520™&530™ ExT Kit-Chef (Thermo Fisher Scientific). Sequencing was performed on Ion GeneStudio S5 (Thermo Fisher Scientific). A maximum of 6 samples were loaded on a single chip per sequencing run.

#### 2.3.3 Bioinformatic analysis

The methylation analysis was performed using the previously described outline (14, 15) and the Ion Torrent Suite™ Software (version 5.10.1) running on the Torrent Server (Thermo Fisher Scientific) was used to process the sequencing data. The “methylation_analysis” Torrent Suite plugin (Thermo Fisher Scientific) was used to analyze the sequencing output of the OPERA_MET-A panel and annotate the percentage of each targeted CpG site. This analysis plugin performs sequencing read alignment onto the W and C strands of the GRCh37/hg19 reference genome and then assesses the methylation status in a strand-specific manner. Reports and text files were generated for each amplicon, containing the number of methylated and unmethylated reads as well as the percentage of methylation per amplicon in relation to the targeted region/CpG sites (14, 15). A summary report was created for each sample that includes the barcode name, the assigned sample name, the total number of reads that cover the target CpGs, and the percentage of methylated reads. In addition, for each barcode, text files with the number of methylated reads, unmethylated reads, and percent methylation for each amplicon were generated. Separate text files were created for (i) the target CpGs, (ii) all CpGs in the amplicon insert, and (iii) all non-CpG cytosines in the amplicon insert. Each amplicon may contain zero, one, or more CpG targets (hotspots) of interest.

## 3 Statistical analysis

A simple linear regression model (Observed methylation ~ Expected methylation), was used to model the relationship between observed and expected global methylation results using the OPERA MET-A panel. The “Expected values” were % of methylation level of different control mixtures obtained from fully methylated and unmethylated commercial gDNA samples (>95%, ~90%, ~75%, ~50%, ~25%, ~10%, <5%). The Wilcoxon signed-rank test was used to assess significant differences in CpG methylation levels between the W and C strands, as defined by genes and patients. All results were deemed statistically significant when *p* is <0.05. R Foundation for Statistical Computing was used to perform all statistical analyses and plots (version 4.0, packages: ggplot2, dplyr, devtools, PairedData).

## 4 Results

### 4.1 Targeted regions selection

The Ion AmpliSeq™ Design Pipeline considered theoretical CpG genome conversion to perform primer design and selection, amplicon tiling and generation of optimal amplicon pooling. The input targets were a list of both single hotspot CpGs (CpG interspersed) and genomic regions containing CpG sites (CpG Island). The target regions of selected genes ranged from 125 to 175bp. They were chosen for validation among those having a potential translational impact in peer-reviewed scientific papers related to immunotherapy in various cancer types (*PD-1, PD-L1*) (16–27), oxidative stress (*KEAP1, NFE2L2, GPX2, PGD, TXNRD1, GCLC, AKR1C2, SRXN1, ABCC2*) (28–35), and other cancer/early-stage prognostic biomarkers (*SPARC, HAR1B, TMPRSS4, RASSF1A, HOXA9, PTEN, CDKN2A/P16*) mainly related to lung cancer (36–59), (**Supplemental Table 2**). For each gene included in the NGS panel, both island-located and interspersed CpGs were tacked (**Table 1**).

**Table 1:** Full list of targeted CpGs by OPERA_MET-A panel.

Target ID	Gene	Accession Number	Chromosome	Start (hg19)	End (hg19)	CpG location (Island/interspersed)	Gene location
**cg04909257**	*PGD*	NM_001304451.2	chr1	10462497	10462499	interspersed	intron
**CpG_Island**	*NFE2L2*	NM_001145412.3	chr2	178128273	178129847	Island	intron/exon
**cg11532131**	*PD-1/CD279*	NM_005018.3	chr2	242792224	242792226	interspersed	exon
**cg10057601**				242793077	242793079	interspersed	exon
**cg22235901**				242793206	242793208	interspersed	exon
**CpG_Island-1**				242794853	242795083	Island	intron/exon
**cg25798782**				242795281	242795283	interspersed	intron
**cg01632474**				242799311	242799313	interspersed	intron
**cg21670983**				242799459	242799461	interspersed	intron
**CpG_Island-2**				242799488	242799696	Island	intron
**cg18096388**				242800972	242800974	interspersed	exon
**cg25890838**				242801045	242801047	interspersed	exon
**cg02122525**				242801251	242801253	interspersed	5’ upstream
**cg14453145**				242801895	242801897	interspersed	5’ upstream
**cg17322655**				242802126	242802128	interspersed	5’ upstream
**cg20805133**				242802191	242802193	interspersed	5’ upstream
**cg19811994**	*RASSF1A*	NM_170713.3	chr3	50373640	50373642	interspersed	intron
**CpG_Island-1**				50374264	50375629	Island	intron/exon
**cg24049629**				50376474	50376476	interspersed	intron
**CpG_Island-2**				50377803	50378540	Island	intron/exon/5’ upstream
**cg10505630**	*SPARC*	NM_003118.4	chr5	151051213	151051215	interspersed	intron
**cg23174201**				151054255	151054257	interspersed	intron
**cg27128761**				151055649	151055651	interspersed	intron
**cg26389330**				151057859	151057861	interspersed	intron
**cg14518209**				151066267	151066269	interspersed	intron
**CpG_Island**				151066456	151066695	Island	exon
**cg25913233**				151066682	151066684	interspersed	5’ upstream
**cg22116670**				151066729	151066731	interspersed	5’ upstream
**cg07539983**				151067340	151067342	interspersed	5’ upstream
**cg02731193**	*GCLC*	NM_001498.4	chr6	53407185	53407187	interspersed	intron
**CpG_Island**	*HOXA9*	NM_152739.4	chr7	27203915	27206462	Island	intron/exon/5’ upstream
**cg15837913**	*PD-L1/CD274*	NM_014143.4	chr9	5449889	5449891	interspersed	5’ upstream
**CpG_Island**				5450409	5450629	Island	intron/exon/5’ upstream
**cg13474877**				5450723	5450725	interspersed	intron
**cg19724470**				5450935	5450937	interspersed	intron
**cg12840719**	*CDKN2A*	NM_000077.5	chr9	21968232	21968234	interspersed	exon
**CpG_Island-1**				21968358	21968728	Island	intron
**CpG_Island-2**				21970913	21971190	Island	exon
**CpG_Island-3**				21974578	21975306	Island	intron/exon
**cg19648686**	*AKR1C2*	NM_001354.6	chr10	5044991	5044993	interspersed	intron
**CpG_Island**	*PTEN*	NM_000314.8	chr10	89621772	89624128	Island	intron/exon*
**cg02307823**				89675900	89675902	interspersed	intron
**cg19378330**	*ABCC2*	NM_000392.5	chr10	101605987	101605989	interspersed	intron
**cg05775918**	*TMPRSS4*	NM_019894.4	chr11	117947554	117947556	interspersed	5’ upstream**
**cg03634928**				117947610	117947612	interspersed	5’ upstream**
**cg27300950**				117947627	117947629	interspersed	5’ upstream**
**cg25116503**				117947656	117947658	interspersed	5’ upstream**
**cg22957898**				117947876	117947878	interspersed	exon**
**cg03331715**	*TXNRD1*	NM_182729.3	chr12	104689086	104689088	interspersed	intron
**cg10880599**	*GPX2*	NM_002083.4	chr14	65408479	65408481	interspersed	intron***
**cg09643186**				65409451	65409453	interspersed	exon***
**cg26155983**				65410144	65410146	interspersed	5’ upstream
**cg01586432**	*KEAP1*	NM_203500	chr19	10597015	10597017	interspersed	exon
**cg02337283**				10599975	10599977	interspersed	exon
**cg22779878**				10600445	10600447	interspersed	exon
**CpG_Island**				10602280	10602878	Island	exon
**cg20226327**				10602959	10602961	interspersed	intron
**cg24892871**				10611042	10611044	interspersed	intron
**cg26988016**				10612801	10612803	interspersed	intron
**cg15204119**				10613179	10613181	interspersed	intron
**cg06911149**				10613455	10613457	interspersed	intron
**cg15676203**				10613487	10613489	interspersed	intron
**cg03890664**				10613491	10613493	interspersed	intron
**cg26500801**				10613854	10613856	interspersed	intron
**cg02428100**				10614021	10614023	interspersed	exon
**cg25801292**				10614271	10614273	interspersed	5’ upstream
**cg12095186**				10615096	10615098	interspersed	5’ upstream
**cg03754063**				10615198	10615200	interspersed	5’ upstream
**cg18484212**	*SRXN1*	NM_080725.3	chr20	631460	631462	interspersed	intron
**CpG_Island**	*HAR1B*	NR_003245.1	chr20	61733275	61734521	Island	intron/exon

*(“intron/exon” for KLLN, NM_001126049.2, C strand);

**(“intron” for SMIM35, NM_001394164.1, C strand);

*** (“intron” for FTNB, NM_001202559.1, W strand).

### 4.2 Samples type and libraries profile – pre-sequencing quality control

**Figure 1:**
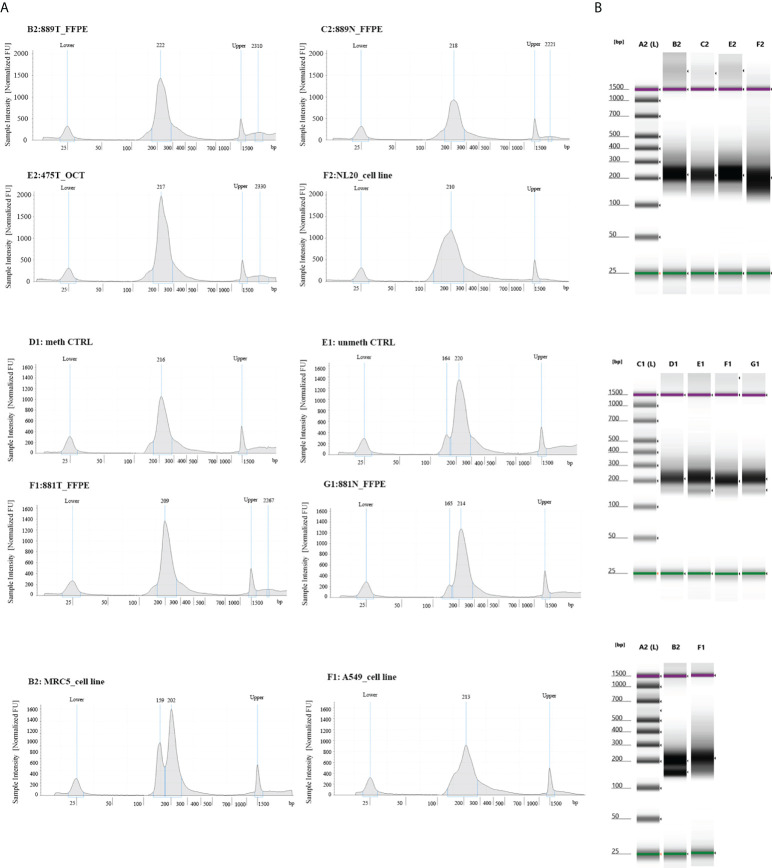
Quality control steps of library construction using the OPERA_MET-A panel for targeted next generation bisulfite sequencing. **(A)** Representative picture of chromatograms showing a high quality library profile of methylated and unmethylated DNA control samples and eight gDNA samples from cell lines, FFPE and OCT embedded tissues. **(B)** Representative images from Agilent Tape Station 2200 NGS libraries for all bisulfite treated DNA samples.

To experimentally validate the performance of our NGS methylation panel, we decided to test three different types of biological samples having different grades of fragmentation and verify whether and how the density of global and single methylation status for each CpG site/region at gene Island occurs. The different epi/methyl-print is based on a set of 3 paired FFPE lung tumors/matched non-neoplastic tissues, 3 OCT embedded lung tumors samples and adenocarcinoma lung cancer cell A549 and non-neoplastic cell MRC5 lines. Upon libraries amplification, the quality and molar concentration of each library were determined using Tape Station 2200 (Agilent) and no substantial differences in the libraries quality and length were observed among converted gDNA templates from cell lines, OCT embedded and FFPE Tissues (**Figure 1**). By contrast, differences in quantity and library profiles were observed when an input of converted gDNA amounts of 30 ng and 100 ng were used to construct libraries (**Supplemental Figure 1**). Considering the concentration and size distribution of amplicons, the higher libraries quality profile was observed for 100 ng of converted gDNA, which was therefore adopted for all the above reported experiments.

### 4.3 Evaluation of analytical performance of OPERA_MET-A panel

Converted gDNA from cell lines, OCT embedded and FFPE tissues were successfully analyzed. Results about metrics per sample was summarized in **Table 2**. The mean number of reads for samples was 1034910 ± 101511 (ranging from 329889 to 2077231), the mean number of read length was 76 ± 2 (ranging from 57 to 85 bp); the mean number of mapped reads was 769384 ± 74468 (ranging from 244244 to 1397720), the mean percentage of read on target was 55 ± 3% for W strand (ranging from 33% to 79%), and 53 ± 4% for C strand (ranging from 23% to 81%). A difference in % of unmapped reads was observed between tested mixes of commercially bisulfite treated gDNA samples and bisulfite treated gDNA obtained from biological sample biopsies (≤12% *vs* ≥26%). Anyway, a high intra-samples variability was observed among gDNA from all samples type (**Supplemental Table 3**). The mean number of read per amplicon was 2862 ± 304 for W strand (ranging from 1035 to 5875), and 3081 ± 345 for C strand (ranging from 333 to 6433), in line with the data previously reported by Luo et al. for the Ion AmpliSeq™ Methylation Panel for Cancer Research (15).

**Table 2:** Metrics per sample obtained using the OPERA_MET-A panel for targeted bisulfite NGS.

Metric	Mean value	Minimum observed	Maximum observed
Number of total reads	1034910	329889	2077231
Number of mapped reads	769384	244244	1397720
Read length (base pair)	76bp	57bp	85bp
Percentage of read on target for W strand	55%	33%	79%
Percentage of read on target for C strand	53%	23%	81%
Number of read per amplicon for W strand	2862	1035	5875
Percentage of read on target for C strand	3081	333	6433

### 4.4 Estimation of optimal samples per chip number

The empirical calculation of cut-off of minimum required mapped reads per target was made according to the following criteria: >20 methylated read for testing CpG site, >40% of tumor cell content and >10% methylation level per site and was also related to an expected conversion efficiency >99%. We can therefore assume a minimum of 500 total mapped reads per site to achieve a successful run/site. Based on the experimental sequencing performance of samples, we assume that a minimum of 6 samples/530 chip can be loaded.

### 4.5 Performance of bisulfite conversion rate and global methylation detection by OPERA_MET-A panel

Bisulfite conversion of gDNA was employed in order to discriminate methylated *versus* unmethylated cytosines, where unmethylated cytosines were deaminated to uracil nucleotide; converted DNA template generated thymines during PCR amplification. The bisulfite conversion rate was calculated by evaluating the Lambda control DNA. Once bisulfite conversion is performed, theoretically every C residue in the unmethylated Lambda control DNA should be converted to a T.

The methylation_analysis plugin counted the number of C residues that are present in the sequence to determine the percentage of the sequence that is methylated, assuming that after the bisulfite conversion reaction, the only C residues that remain in the sequence were methylated in the original sample. The value is shown in the percent.ME (percent methylation) column for each sample. The optimal bisulfite conversion rate should be >99%, calculated as the difference from 100% of the average of the observed W and C percent.ME values for the unmethylated Lambda DNA in each sample and control (60).

To estimate the conversion efficiency of our samples, replicate commercially gDNA samples of average methylation states across all CpGs of approximately >95% and <5%, and different mixtures of the two methylation states (~90%, ~75%, ~50%, ~25% and ~10%) were used to construct libraries using the custom panel. As per standard operative procedure, unmethylated Lambda DNA was spiked into each sample prior to bisulfite conversion and primers exist in the panel to determine conversion efficiency using the sequencing output files. A high conversion efficiency was obtained for all treated control samples, with a mean of 98,5% ± 0,2 of conversion efficiency (ranging from 97,5% to 99,1%), (**Supplemental Figure 2**).

**Figure 2:**
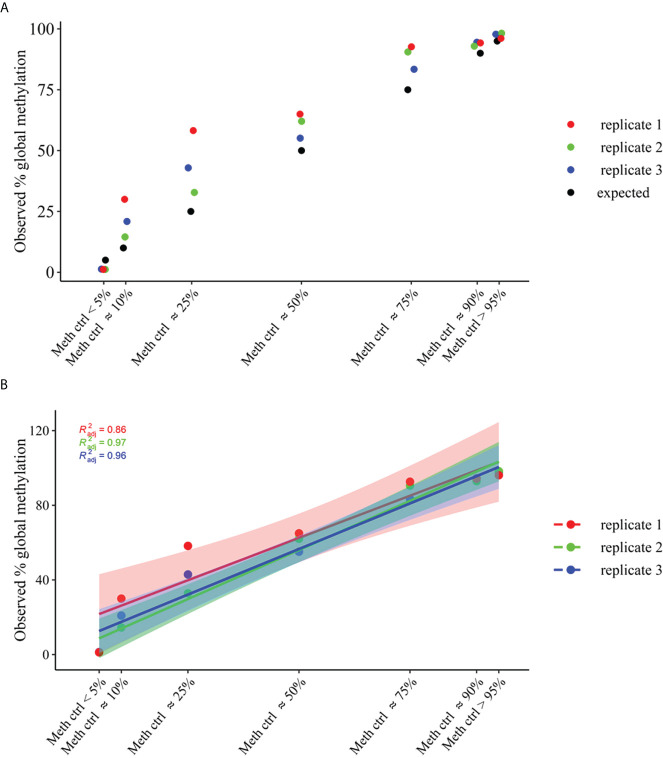
Global methylation levels across all amplicons (target_CpGs) of OPERA_MET-A panel. gDNA samples of average methylation states across all CpGs of approximately >95% and <5%, and different mixtures of methylation states (~90%, ~75%, ~50%, ~25% and ~10%) were used. **(A)** Distribution of observed global methylation levels for each gDNA control mixture in three replicates (replicate 1=red, replicate2=green, replicate3=blue), the expected ones are in black. **(B)** Linear regression analysis of global methylation levels for each gDNA control mixtures (replicate1=red, replicate2=green, replicate3=blue). The filled color areas represent the standard error of each replicate.

The methylation_analysis plugin allowed both the alignment and the methylation status calling. As a result, a high concordance between average percent methylation across all amplicons (target_CpG) of OPERA_MET-A panel between observed and expected % of methylation was obtained for each methylation state (*p value*<0.0001, Adjusted R2 = 0.95), (**Figure 2**).

**Figure 3:**
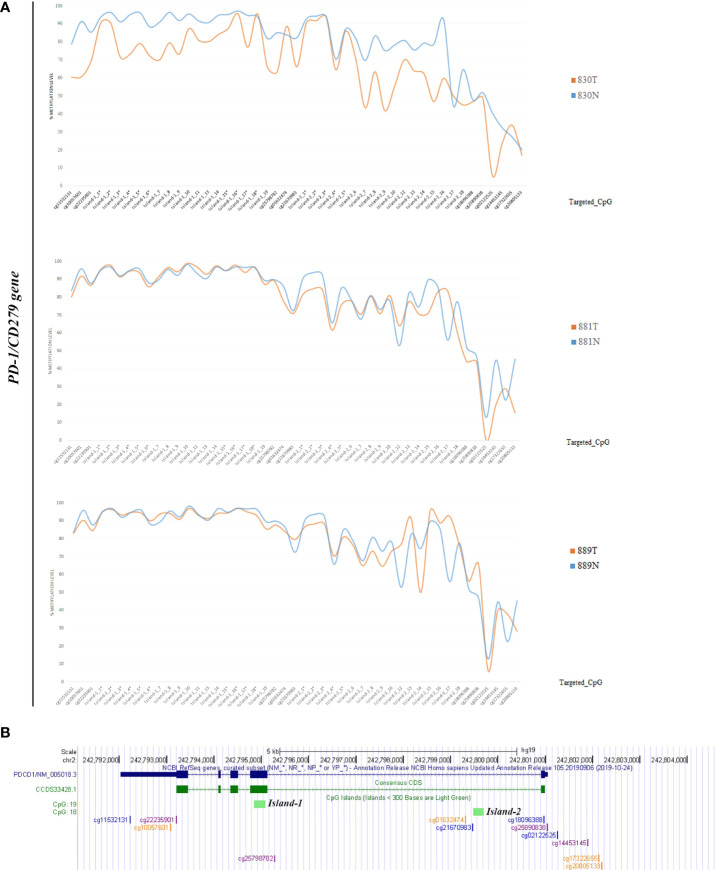
**(A)** Schematic epigrams showing the CpGs methylation levels along different *PD-1/CD279* gene regions in paired tumor/normal FFPE samples. **(B)** Schematic representation of the *PD-1/CD279* gene structure within the human hg19 genome sequence. From top to bottom: NCBI RefSeq and Consensus CDS tracks for *PD-1/CD279* exon/intron structure; predicted CpG islands (“Regulation” >> “CpG Island” track); CpG methylation sites map targeted by OPERA_MET-A panel are mapped.

**Figure 4:**
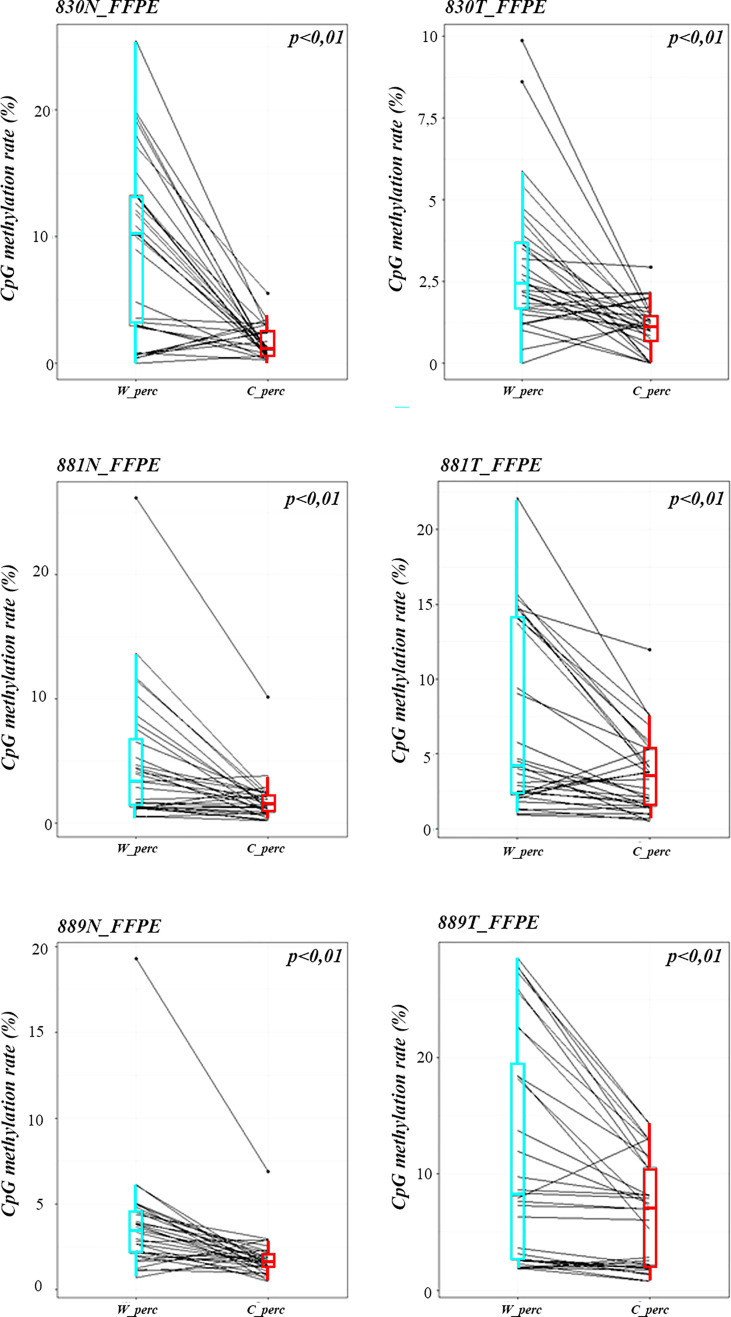
Strand specific distributions of CpG methylation in samples using OPERA_MET-A panel. The average percent of global CpG methylation at *RASSF1A* gene (target_CpGs) in paired non-neoplastic and tumor tissues of FFPE samples 830, 881, 889. Global % CpG methylation at W strands is in light blue boxplots, at C strands in red boxplot (*Wilcoxon signed-rank test*).

Methylation data obtained by NGS from FFPE samples (paired tumor/non-neoplastic samples) were used to evaluate the utility of OPERA_MET-A panel to visualize the CpG methylation distribution along different gene regions (Island and interspersed CpGs). As expected, variations of methylation level of CpGs were observed between paired samples from the same patients and among patients for *PD-1/CD279*, that was shown as representative model for this specific application (**Figure 3**). Finally, methylation data obtained by NGS from paired FFPE samples to investigate the CpG methylation distribution between W and C strands. A subset of CpGs mapped in *KEAP1*, *PD-1/CD279* and *RASSF1A* genes (**Supplemental Table 1**) was chosen as pilot for this analysis. For each targeted CpG located on both W and C strand, the total number of reads that cover the target CpGs, the number of methylated reads, unmethylated reads, and percent methylation for each amplicon were generated for each targeted CpG site on both W and C strand of the genes. Interestingly, we observed a striking change in global CpG methylation across the *RASSF1A* gene on both W and C strands in all samples (p<0.01, Wilcoxon signed-rank test), whereas no substantial differences in global CpG methylation distribution between W and C strands were observed in *CD279*/*PD-1* and *KEAP1* genes (**Figure 4** and **Supplemental Figures 3**, **4**).

## 5 Discussion

With an increasing number of clinically actionable targets, the optimization of NGS technology ensured a high sensitivity, specificity and time-saving of analysis by easily assessing every nucleotide change in multiple targets gene/or regions at single-base resolution. More recently, the NGS technology is also enhancing the methylome analysis, thus contributing to expand the knowledge and characterization of differentially methylated gDNA regions in many cancer-related human genes (61). Starting from a relatively low quantity of biological materials, the NGS approach offers in this specific field the great advantage to successfully quantify DNA methylation density and its differences at specific CpG sites of both promoter or intragenic regions (62, 63). The great main translational advantage of this feasible high-throughput approach in a more specific cancer context is the opportunity to construct a more robust and integrated algorithm to predict the disease evolution of patients. Moreover, it could support the selection and a better stratification of surgically-resected patients for follow-up and enhance the development of novel therapeutic approaches, not yet based on an epigenetic profile in daily practice (64, 65).

The design and validation of the OPERA_MET-A panel for NGS analysis were based on this assumption. This panel allows to obtain libraries starting from gDNA extracted from different matrices, such as cell lines, OCT embedded frozen and FFPE tissues. Considering the recently published data on NGS for methylation analysis using the gene-targeted AmpliSeq technology, very promising and comparable results to those already been published were obtained (15) in terms of mean target depth ≥2,500X (W and C strand), average number of mapped reads >750,000/sample and concordance results between expected and observed % of global methylation for all CpGs. More specifically, the OPERA_MET-A panel primer design pipeline include more amplicons (155 amplicons) than those generated by Ion Ampliseq™ Methylation Panel for Cancer Research (40 amplicons) that covers 18 non-overlapping genes and can be used to analyze gDNA from cell lines, human FFPE and OCT tissues to reach a comparable performance in terms of time consuming, observed/expected methylation rate concordance and average mapped reads. The same “methylation_analysis” plugin was used to successfully perform alignment and methylation calling for amplicons on both W and C strands.

**Figure 5:**
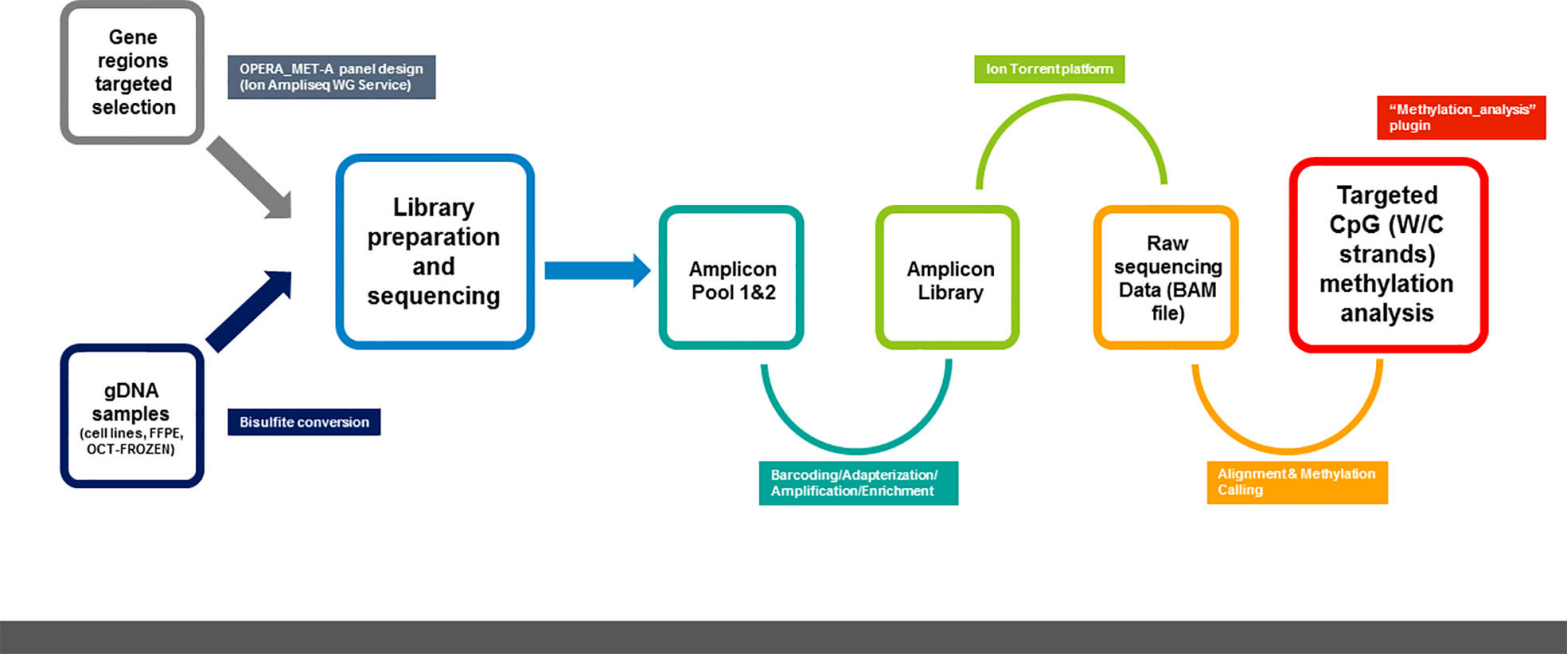
Schematic representation of methylation NGS analysis workflow using OPERA_MET-A panel.

Moreover, the values of the obtained run metric parameters confirm that our customized panel allows us to obtain targeted and quantitative information on tumor markers with a high resolution starting from a relatively low DNA quantity and quality input (**Figure 5**).

The OPERA_MET-A panel covers multiple regions of 18 cancer-driver genes whose methylation profile was previously proven to have a translational impact on lung cancer progression but also in other solid tumors. Among these, the panel allows the epigenetic scanning of poorly investigated CpGs located at the intragenic exon 3 island of the *KEAP1* gene, that were linked to *KRAS* mutant status in NSCLC patients (29) and at the *SPARC* gene promoter, which has been proposed as an interesting prognostic biomarker in NSCLC with useful application in the squamous early-stage group (40). Moreover, CpGs located at the *CD274/PD-L1* promoter region and *CD279/PD-1* were also included in the panel design, since they have recently emerged as an independent prognostic factor associated with shorter overall survival in triple negative breast, colorectal, prostate, gastric, papillary thyroid, melanoma and head and neck cancer patients (16–27). Many increasing literature evidences suggest that, not only CpG Islands, but also CpG shores methylation correlate with gene expression (66, 67). Furthermore, additional evidence would suggest methylation regulatory regions can extent into exon1/intron 1 of a gene and are outside the promoter/CpG island locus (68–70). In the presented work, we mainly focused on CpG island loci and selected the candidate gene list and loci based on an existing literature evidences and own research field of interest results (**Supplemental Table 2**). Further planned analyses will evaluate differences in prognostic impact related to CpGs in single gene or among genes of the presented panel in cancer patients, thus allowing an upgrade of OPERA_MET-A panel.

While the main focus of this study was to investigate methylation on multiple cancer-related genes by using a multigene NGS panel starting from low DNA quality and quantity, our analysis also offers new insight into the heterogeneity of CpG methylation patterns among genes and patients. Using the “methylation_analysis” plugin to manage data obtained from the OPERA_MET-A panel, differentially methylated cytosines can be found along the same gene (i.e. *PD-1/CD279*) and/or in each gene in both double-stranded (i.e., symmetric, as for *PD-1* and *KEAP1* genes) and single-stranded (i.e., asymmetric, as for *RASSF1A*) contexts. While single stranded DNA methylation can be more frequently detected in non-CG methylation contexts (71), CpG positions are usually expected to be either fully methylated or fully unmethylated in both DNA strands. Therefore, there should not be a “right” or “wrong” strand when choosing to study conventional methylation patterns and data obtained from one strand can be safely assumed to apply also to the second strand. Double stranded DNA methylation primarily occurs in the case of nucleobase symmetry between sense (CG) and antisense (GC) DNA strands. However, methylation in a CG context is not always double-stranded; it can also occur on just a single strand, as described in solid tumors (72) and should be related to genes function and transcription. By consequence, the ability to determine the exact locations and status of CpG methylation in W and C strands separately could provide interesting experimental evidence for innovative clinical applications.

The main limitation of the OPERA_MET-A panel relies on the limited number of genes and samples to be analyzed in a single run to achieve an acceptable coverage to obtain epigenetic information from degraded samples having variable % tumor cell content.

Further improvements are required, such as extending the number of clinically relevant genes whose methylation has a prognostic value in tumors and additional orthogonal evaluation of the methylation density using conventional approaches to study methylation of genes at single CpG level. It should also keep in mind that, when we worked on different matrices, including fixed tissues, an optimized pre-analytical and analytical workflows are demanded in order to obtain an optimal quality and quantity of DNA and decrease the risk of suffering from a critical bisulfite conversion. As also reported in our small subsets of samples from different matrices (commercial gDNA, cell lines, OCT and FFPE tissues) degraded DNA samples (by cross-linking, deamination and fragmentation) could in fact affect the efficiency of NGS analysis, so their pre-analytical and analytical manipulation represents a critical aspect to evaluate in order to improve the homogeneity and efficiency of bisulfite conversion and high throughput NGS sequencing (73, 74).

In conclusion, considering all together, the obtained NGS performance for OPERA_MET-A panel corroborates the utility NGS approach for methylation pattern analysis among groups, validation of whole approaches, identification of gDNA methylation for different regions of single/multiple genes (promoter and other regulatory regions) or CpG islands in multiple samples aimed at the discovery of biomarkers having clinical relevance.

## Data availability statement

The original contributions presented in the study are included in the article/**Supplementary Material**. Further inquiries can be directed to the corresponding authors.

## Ethics statement

The studies involving human participants were reviewed and approved by Local Ethical Committee of IRCCS Casa Sollievo della Sofferenza Hospital, Italy. Written informed consent for participation was not required for this study in accordance with the national legislation and the institutional requirements.

## Author contributions

Conceptualization, supervision, writing—original draft preparation, LAM. Methodology and validation FF, FC and MC. Visualization, formal analysis and software SC, TM and MM. Data curation, FF, AS, LAM. Resources, NC and PG, and Investigations, FF, SC, FC. Writing—review and editing: FF, SC, MM, DT, AC, PG, EM, VF, LAM. Funding acquisition, LAM and EM. All authors have read and agreed to the published version of the manuscript.

## Funding

This research was funded by the Italian Ministry of Health, Ricerca Corrente 2021-22, by the “5 x1000” voluntary contributions to Fondazione IRCCS Casa Sollievo della Sofferenza.

## Acknowledgments

The authors thank Dr. Giorgio Pea for his technical and logistic assistance in the OPERA_MET-A panel design; NGS run parameters and Methylation_analysis plugin optimization. The authors also thank Prof. Andreina Guarnieri for the professional English editing of the manuscript, Teresa Balsamo for her technical assistance in performing NGS analysis and Federica Russo for tissue samples handling.

## Conflict of interest

The authors declare that the research was conducted in the absence of any commercial or financial relationships that could be construed as a potential conflict of interest.

## Publisher’s note

All claims expressed in this article are solely those of the authors and do not necessarily represent those of their affiliated organizations, or those of the publisher, the editors and the reviewers. Any product that may be evaluated in this article, or claim that may be made by its manufacturer, is not guaranteed or endorsed by the publisher.

## Supplementary material

Supplementary Figure 1 Input of converted DNA comparison for libraries construction using the OPERA_MET-A for targeted next generation bisulfite sequencing. **(A)** Representative picture of chromatograms showing library profiles starting from 100ng (D1, E1, F1, G1) and 30ng (H1, A2, B2, C2) of input DNA respectively. **(B)** Representative images from Agilent Tape Station 2200 NGS libraries for all bisulfite treated DNA samples.

Supplementary Figure 2 Conversion efficiency across methylated/unmethylated control mixtures using OPERA_MET-A panel. Commercially control DNA samples with average methylation of approximately >95% and <5%, and different mixtures of methylation states (~90%, ~75%, ~50%, ~25% and ~10%) were evaluated in triplicate. The bisulfite conversion efficiency was evaluated by using the Lambda control DNA, that was added before starting the bisulfite conversion to each single DNA. R1, replicate 1, R2, replicate 2, R3, replicate 3.

Supplementary Figure 3 Strand specific distributions of CpG methylation in samples using OPERA_MET-A panel at *CD279/PD-1* gene. The average percent of global CpG methylation (target_CpGs) in paired non-neoplastic and tumor tissues of FFPE samples 830, 881, 889. Global % CpG methylation at W strands is in light blue boxplots, at C strands in red boxplot (Wilcoxon signed-rank test).

Supplementary Figure 4 Strand specific distributions of CpG methylation in samples using OPERA_MET-A panel at *KEAP1* gene. The average percent of global CpG methylation (target_CpGs) in paired non-neoplastic and tumor tissues of FFPE samples 830, 881, 889. Global % CpG methylation at W strands is in light blue boxplots, at C strands in red boxplot (Wilcoxon signed-rank test).
