# Supplementary figures and images for: Design and experimental validation of OPERA_MET-A panel for deep methylation analysis by next generation sequencing
Source: Front Oncol. 2022 Aug 11;12:968804. doi: 10.3389/fonc.2022.968804 (PMC9404304; doi:10.3389/fonc.2022.968804)

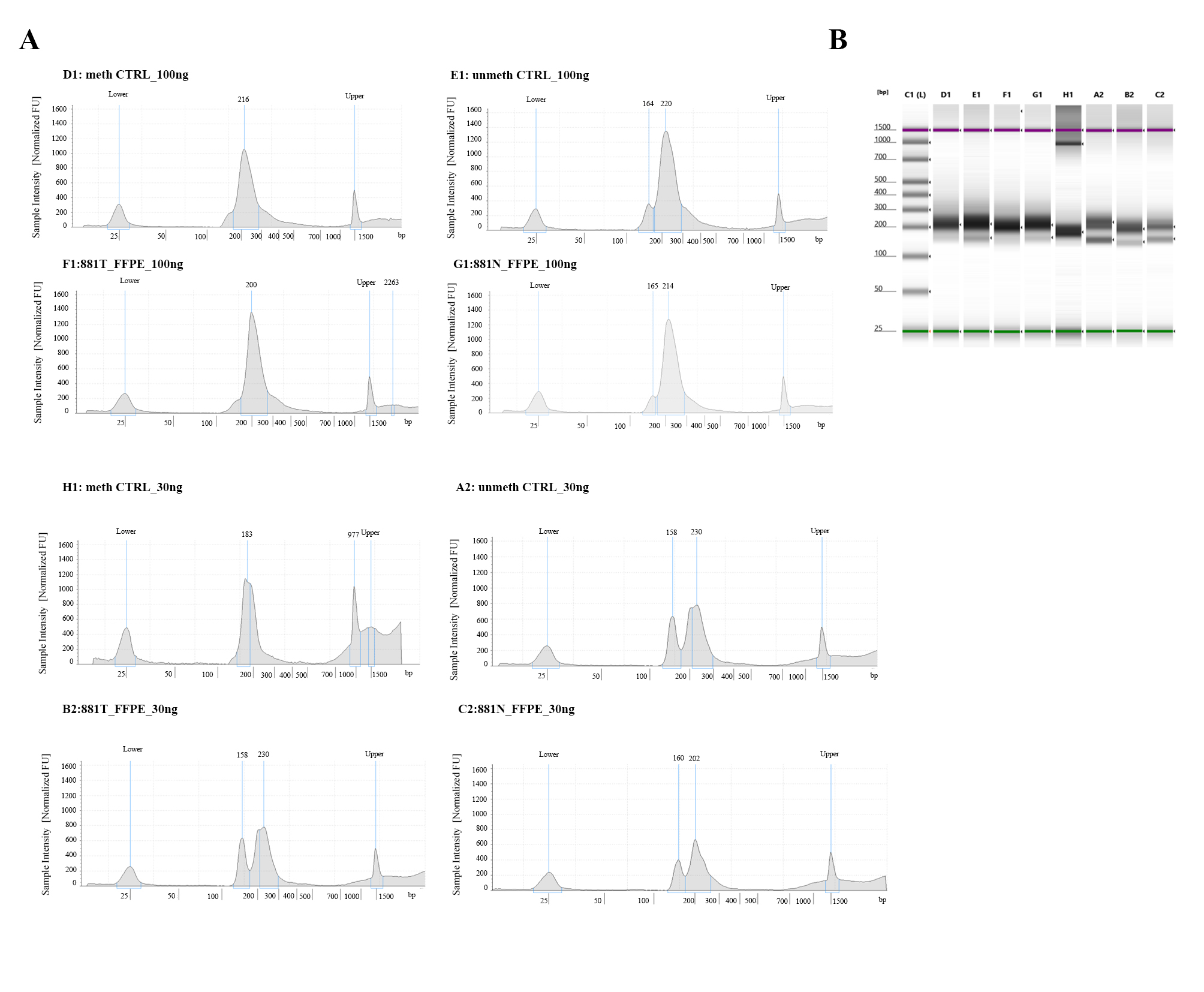

Supplement: Supplementary Figure 1 — Input of converted DNA comparison for libraries construction using the OPERA_MET-A for targeted next generation bisulfite sequencing. (A) Representative picture of chromatograms showing library profiles starting from 100ng (D1, E1, F1, G1) and 30ng (H1, A2, B2, C2) of input DNA respectively. (B) Representative images from Agilent Tape Station 2200 NGS libraries for all bisulfite treated DNA samples. [file Image_1.jpeg]

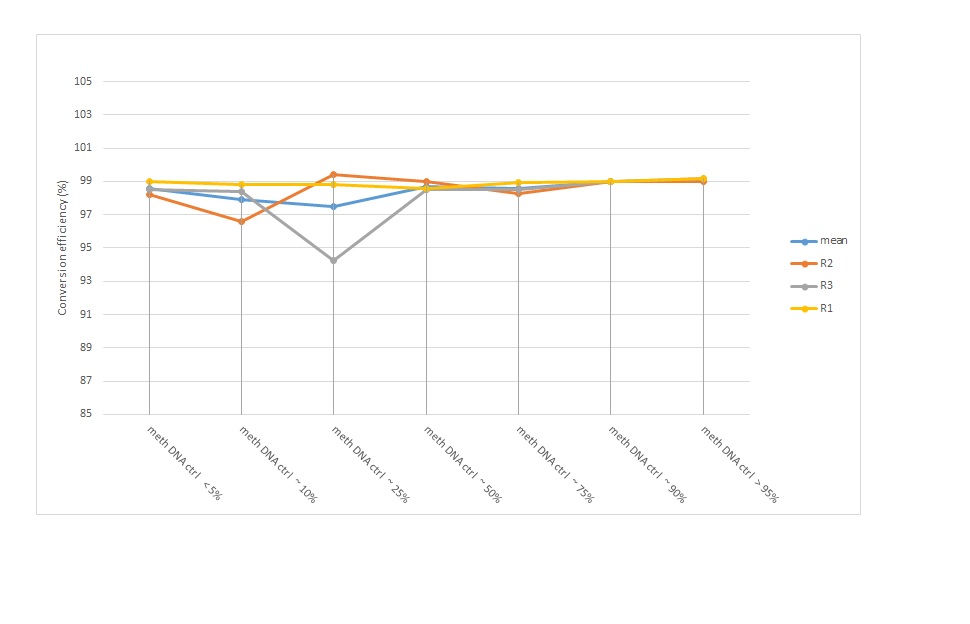

Supplement: Supplementary Figure 2 — Conversion efficiency across methylated/unmethylated control mixtures using OPERA_MET-A panel. Commercially control DNA samples with average methylation of approximately >95% and <5%, and different mixtures of methylation states (~90%, ~75%, ~50%, ~25% and ~10%) were evaluated in triplicate. The bisulfite conversion efficiency was evaluated by using the Lambda control DNA, that was added before starting the bisulfite conversion to each single DNA. R1, replicate 1, R2, replicate 2, R3, replicate 3. [file Image_2.jpeg]

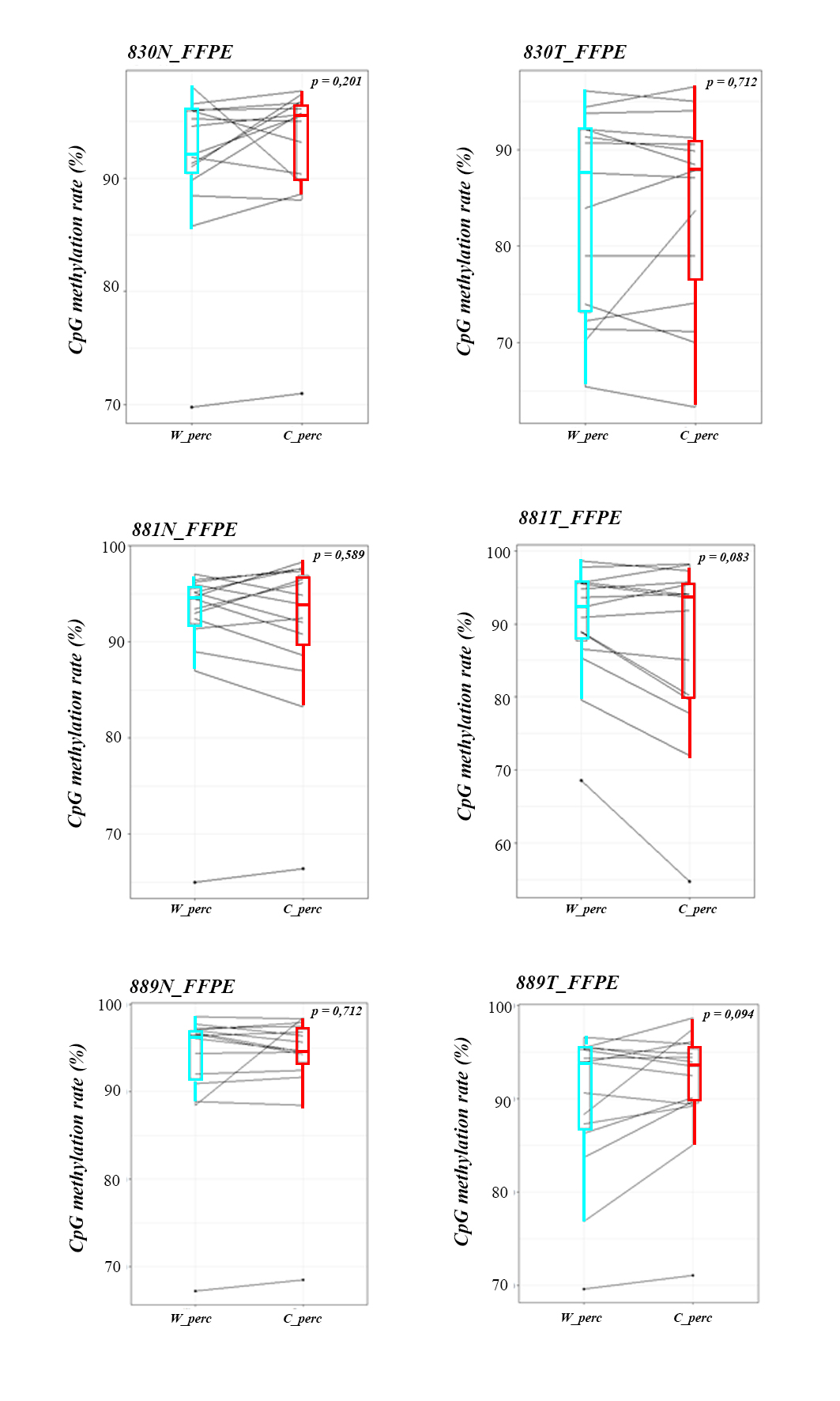

Supplement: Supplementary Figure 3 — Strand specific distributions of CpG methylation in samples using OPERA_MET-A panel at CD279/PD-1 gene. The average percent of global CpG methylation (target_CpGs) in paired non-neoplastic and tumor tissues of FFPE samples 830, 881, 889. Global % CpG methylation at W strands is in light blue boxplots, at C strands in red boxplot (Wilcoxon signed-rank test). [file Image_3.jpeg]

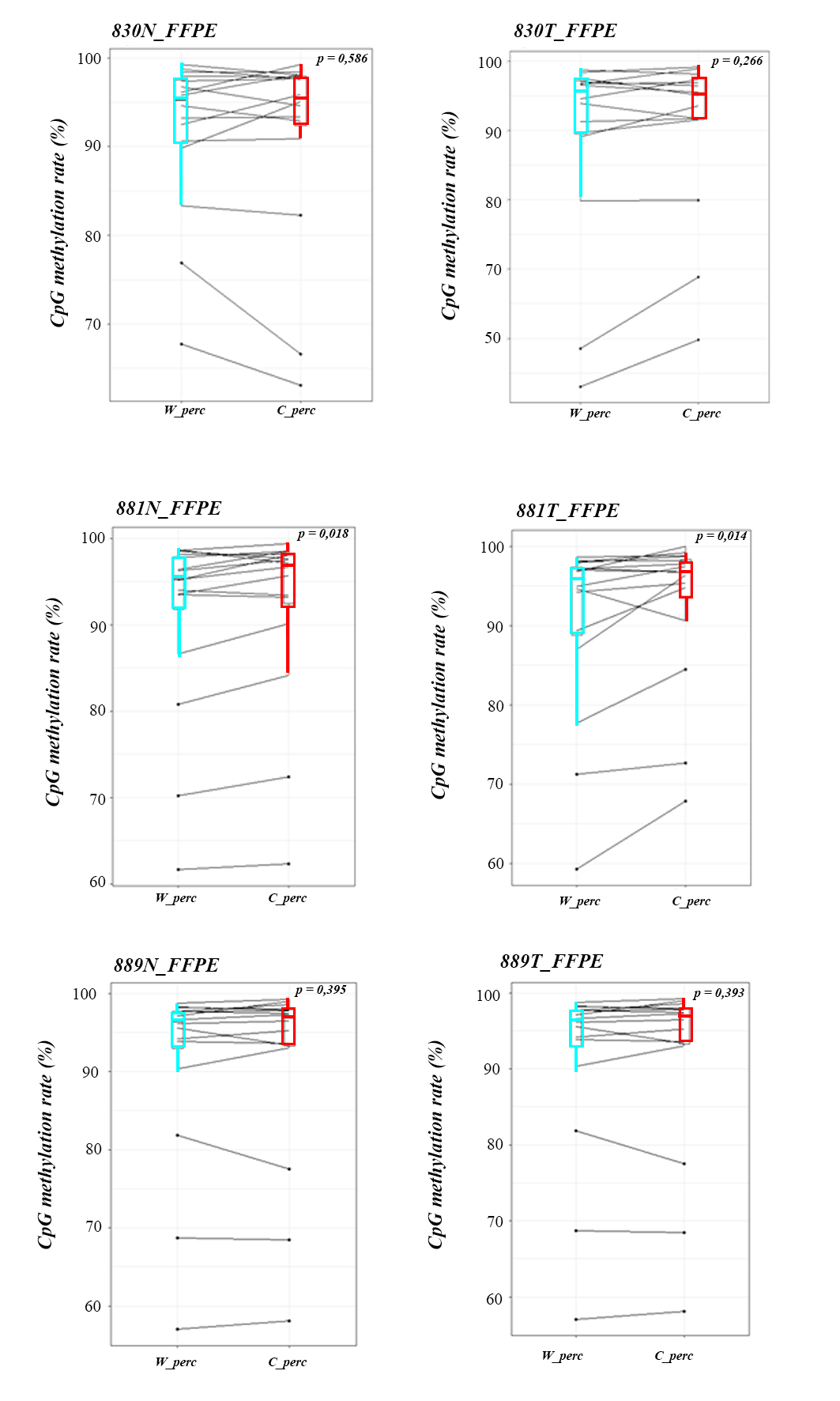

Supplement: Supplementary Figure 4 — Strand specific distributions of CpG methylation in samples using OPERA_MET-A panel at KEAP1 gene. The average percent of global CpG methylation (target_CpGs) in paired non-neoplastic and tumor tissues of FFPE samples 830, 881, 889. Global % CpG methylation at W strands is in light blue boxplots, at C strands in red boxplot (Wilcoxon signed-rank test). [file Image_4.jpeg]
