# Supplementary material for: Design and experimental validation of OPERA_MET-A panel for deep methylation analysis by next generation sequencing
Source: Front Oncol. 2022 Aug 11;12:968804. doi: 10.3389/fonc.2022.968804 (PMC9404304; doi:10.3389/fonc.2022.968804)
Supplement: Supplementary file 6 [file Table_2.docx]

**Supplemental Table 2.** List of genes included in OPERA_MET-A panel and their prognostic impact on cancer patients’ outcomes.

| Gene Symbol | Main Cancer model | REFERENCES |
| --- | --- | --- |
| *PD1/CD279* | Head and neck cancer, Glioma  Thyroid cancer  Merkel cell carcinoma, Melanoma  Lung cancer | **[16-24]** |
| *PD-L1/CD274* | Lung cancer, Melanoma  Gastric cancer, Glioma  Colorectal cancer  Head and neck cancer, Prostate cancer | **[20, 25-27]** |
| *KEAP1* | Lung cancer, Renal cancer  Colorectal cancer, Breast cancer  Glioma, Hepatocellular carcinoma | **[28-30]** |
| *NFE2L2* | Prostate cancer, Colon cancer | **[28, 31, 32]** |
| *GPX2* | Lung cancer | **[33]** |
| *PGD* | Lung cancer | **[33]** |
| *TXNRD1* | Hepatocellular carcinoma  Lung cancer | **[33-34]** |
| *GCLC* | Lung cancer | **[33]** |
| *AKR1C2* | Acute myeloid leukemia | **[35]** |
| *SRXN1* | Lung Cancer | **[33]** |
| *ABCC2* | Lung cancer | **[33]** |
| *SPARC* | Lung cancer, Prostate cancer  Gastrointestinal cancer  Pancreatic cancer,  Hepatocellular carcinoma  Breast cancer, Prostate cancer | **[36-41]** |
| *HAR1B1* | Glioma | **[42]** |
| *TMPRSS4* | Lung cancer | **[43,44]** |
| *RASSF1A* | Lung cancer, Colorectal cancer  Brest cancer, Esophageal carcinoma  Renal cancer, Thyroid cancer | **[45-48]** |
| *HOXA9* | Lung cancer, Head and Neck cancer  Breast cancer, Bladder cancer | **[45, 49-52]** |
| *PTEN* | Lung cancer, Breast cancer | **[53-54]** |
| *CDKN2A* | Lung cancer, Hepatocellular carcinoma  Colorectal cancer, Breast cancer | **[55-59]** |
