# Supplementary material for: Design and experimental validation of OPERA_MET-A panel for deep methylation analysis by next generation sequencing
Source: Front Oncol. 2022 Aug 11;12:968804. doi: 10.3389/fonc.2022.968804 (PMC9404304; doi:10.3389/fonc.2022.968804)
Supplement: Supplementary file 7 [file Table_3.docx]

**Supplemental Table 3.** DNA quality and unmapped reads details of commercial and non-commercial samples amplified using the OPERA_MET-A NGS panel.

| **Sample type** | **Sample name** | **DIN** | **Unmapped reads (%)** |
| --- | --- | --- | --- |
| Commercial gDNA | M ctrl gDNA (>98%) | 6,8* | 0,07 |
| Commercial gDNA | UM ctrl gDNA (<5%) | 6,6* | 0,06 |
| Commercial gDNA | UM ctrl 10%+M ctrl 90% |  | 0,12 |
| Commercial gDNA | UM ctrl 25%+M ctrl 75% |  | 0,13 |
| Commercial gDNA | UM ctrl 50%+M ctrl 50% |  | 0,11 |
| Commercial gDNA | UM ctrl 75%+M ctrl 25% |  | 0,12 |
| Commercial gDNA | UM ctrl 90%+M ctrl 10% |  | 0,26 |
|  |  |  |  |
| OCT human tissue | 435T_OCT | 1,1 | 0,62 |
| OCT human tissue | 475T_OCT | 1,2 | 0,49 |
| OCT human tissue | 495T_OCT | 1,2 | 0,02 |
|  |  |  |  |
| cell line | A549 | 8,9 | 0,44 |
| cell line | MRC5 | 9,3 | 0,25 |
|  |  |  |  |
| FFPE tissue | 830T_FFPE | 4,1 | 2,71 |
| FFPE tissue | 830N_FFPE | 3,1 | 2,70 |
| FFPE tissue | 881T_FFPE | 4,4 | 8,45 |
| FFPE tissue | 881N_FFPE | 3,1 | 50,48 |
| FFPE tissue | 889T_FFPE | 3,1 | 45,04 |
| FFPE tissue | 889N_FFPE | 3,1 | 46,04 |
|  |  |  |  |

*DIN, DNA integrity number *DIN was evaluated only for the fully M and UM ctrls, that were used to obtain each listed ctrl mixes.*

*gDNA, genomic DNA*

*FFPE, formalin fixed paraffin embedded*

*OCT, optimal cutting temperature compound*

*M ctrl, Methylated control*

*UM ctrl, Unmethylated control,*
